# Distribution Evaluation of Tenofovir in the Breast Milk of Mothers With HBeAg-Positive Chronic HBV Infection After Treatment With Tenofovir Alafenamide and Tenofovir Disoproxil Fumarate by a Sensitive UPLC-MS/MS Method

**DOI:** 10.3389/fphar.2021.734760

**Published:** 2021-08-13

**Authors:** Na Yang, Guanlun Zhou, Xiaoliang Cheng, Jun He, Yan Chen, Chao Chen, Meijuan Li, Jiajia Ge, Min Wang, Tianqi Zhang, Weihong Ge, Huaijun Zhu, Guorong Han

**Affiliations:** ^1^ Department of Pharmacy, Nanjing Drum Tower Hospital, The Affiliated Hospital of Nanjing University Medical School, Nanjing, China; ^2^ Department of Obstetrics and Gynecology, The Second Hospital of Nanjing, Nanjing University of Chinese Medicine, Nanjing, China; ^3^ Nanjing Qlife Medical Technology Co., Ltd, Nanjing, China

**Keywords:** tenofovir alafenamide, tenofovir, breast milk, UPLC—MS /MS, HBV

## Abstract

Tenofovir alafenamide (TAF) is a novel prodrug of tenofovir (TFV) that has been approved for the treatment of chronic hepatitis B virus (HBV) infection. It has greater plasma stability and more favorable renal safety than tenofovir disoproxil fumarate (TDF), the first approved oral prodrug of TFV. However, the distribution of TFV in the breast milk of mothers treated with TAF is still unclear. In this study, sixteen participants with chronic HBV infection were enrolled and received antiretroviral therapy with 25 mg of TAF or 300 mg of TDF daily from 24 to 28 weeks of gestation until the 4th week postpartum. For the first time, the distribution of TFV in the breast milk of mothers with chronic HBV infection treated with TAF and its difference from TDF were evaluated by using a sensitive UPLC–MS/MS method. Chromatographic separation was achieved on a Waters ACQUITY UPLC BEH C18 column (1.7 µm 2.1 × 100 mm). Mass spectrometry analysis was performed in positive electrospray ionization mode and multiple reaction monitoring (MRM) conditions of transitions m/z 288.1→176.2 for TFV. This method was linear from 0.5 to 500 ng/ml. Surprisingly, on the third postpartum day, the median Cmax of TFV in the breast milk was much higher in the mothers treated with TAF (101.2 ng/ml) than TDF (21.6 ng/ml) at a similar Tmax of 4 h. Accordingly, the median AUC0-8 value was 755.6 ng h/mL in the mothers taking TAF, which was at a 5-fold higher level than TDF. The concentration of TFV in the breast milk of mothers in both groups decreased with increasing lactation time. These data indicated that there was a relatively higher exposure of TFV in the breast milk of mothers taking TAF, despite the lower dosage compared to TDF. This study provides support for further evaluating the safety of breastfeeding after the administration of TAF and TDF.

## Introduction

Chronic hepatitis B virus (HBV) infection is currently a major global public health problem that causes enormous economic and social burdens (Stanaway et al., 2016). A total of 257 million people are estimated to be infected with HBV according to The 2017 World Health Organization (WHO) Global Hepatitis Report, which means that 3.5% of the world population has chronic HBV infection (WHO, 2017). Despite existing resources for vaccination and treatment, the burden of chronic HBV remains significant within many countries (Shan et al., 2018; Wang et al., 2019). Perinatal or early childhood transmission may account for more than one third of chronic infections, since newborns have a 90% chance of becoming chronic carriers after infection with HBV, while among adults the chance is only up to 5% (Nunes et al., 2014). In China, the rate of HBV infection among women of childbearing age is at a high level (8.16%) (Han et al., 2012; Sheng et al., 2018). For these reasons, preventing mother -to- child transmission (MTCT) of HBV during pregnancy or the perinatal period has been targeted as the most important phase for the prevention of chronic HBV infection. Although immunoprophylaxis with hepatitis B vaccine and hepatitis B immunoglobulin (HBIG) in neonates can remarkably reduce HBV infection (Han et al., 2011; Cheung et al., 2013), it is still far from completely eradicating MTCT of HBV, particularly in those born to mothers with HBeAg positivity and high viremia, which means that high HBV DNA levels and HBeAg positivity in pregnant women are considered as the most important risk factors for MTCT of HBV(Wen et al., 2013; Keane et al., 2016; Boucheron et al., 2021). Consequently, the WHO recommends antiviral therapy administered during pregnancy for the prevention of MTCT (WHO, 2020). Nucleoside analogs (NAs) inhibit HBV-DNA replication and are currently approved for anti-HBV treatment.

Previous study (Han et al., 2011) have proven the efficacy of telbivudine (LdT) administration at 20–32 weeks of pregnancy in blocking mother-to-child transmission of HBV, which has been approved by the FDA for blocking vertical transmission of HBV. However, the drug resistance barrier of LdT is low (Cai et al., 2017). Since its approval, additional studies have shown that anti-HBV NA administration during pregnancy can significantly reduce MTCT of HBV(Tavakolpour et al., 2018; Funk et al., 2021). Among them, tenofovir (TFV) is the only approved NA with high efficacy against the virus and no detected clinical resistance to date, and tenofovir disoproxil fumarate (TDF) treatment, a pro-drug of TFV, has been recommended by the WHO to treat chronic HBV infection (WHO, 2015) since 2015, particularly in pregnant women (Lin et al., 2018; Bierhoff et al., 2020). TDF received US FDA approval for HIV in 2001 first, and approval for HBV in 2008, administered at a dose of 300 mg once daily.

Tenofovir alafenamide (TAF) is a new prodrug of TFV approved by the US FDA in 2015 for the treatment of HBV. After oral administration, some TDF is hydrolyzed by gut and plasma esterase to TFV, but TAF is stable in the plasma and metabolized mostly intracellularly by cathepsin A to TFV (Birkus et al., 2008; Wassner et al., 2020). The pharmacokinetics of TAF led to a 6.5-fold higher intracellular concentration of the active form, TFV diphosphate, with a 91% lower serum concentration of TFV compared to TDF (Ray et al., 2016; Vitoria et al., 2016; Hill et al., 2018). TAF is more stable in plasma and is selectively cleaved into its active metabolite intracellularly, so TAF at a dose of 25 mg can obtain antiviral effects equal to 300 mg TDF, thereby reducing the plasma concentration of TFV, which is associated with high risks of renal and bone adverse events (Gupta et al., 2019).

Despite the accumulating evidence on the efficacy and safety of the two TFV prodrugs during pregnancy, their safety during breastfeeding has not been well studied. As a result, there are inconsistent recommendations on whether HBV-infected mothers who are on TFV treatment should breastfeed or not. Breastfeeding is not discouraged when the mother is on TDF treatment according to the US guidelines (Terrault et al., 2018), but the guideline established by the Chinese Society of Hepatology in China (Hou et al., 2017) states no clear instructions about breastfeeding by HBV-infected mothers who are on TDF treatment, nor is there any recommendation on the drug label of TDF. Limited research has shown a relatively lower (3.2–14.1 ng/ml) maximum TFV concentration in the breast milk of mothers treated with a 300 mg daily dose (Benaboud et al., 2011; Mugwanya et al., 2016; Waitt et al., 2018), but no similar studies have been published for TAF.

In this study, a sensitive and robust ultra-performance liquid chromatography–tandem mass spectrometry (UPLC–MS/MS) method was established and validated for the quantification of TFV in human breast milk. For the first time, we estimated and compared the distribution of TFV in the breast milk of mothers with HBeAg-positive chronic HBV infection during treatment with TAF and TDF, two prodrugs of TFV. This study provides support for further evaluating the safety of breastfeeding after administration of TAF and TDF.

## Materials and Methods

### Chemicals and Materials

TAF and TDF were provided by Gilead Sciences, Inc.(California, United States) and GlaxoSmithKline (Ware, United Kingdom), respectively. TFV (purity 99.1%) and TFV-d6 (purity 95.0%) standards were purchased from TRC (Toronto, Canada). Acetonitrile (HPLC grade), ammonium acetate and formic acid were purchased form Merck (Darmstadt, Germany) or Fisher (Waltham, MA, United States). Deionized water was prepared using a Milli-Q system (Millipore, Milford, MA, United States). All other reagents were commercially available.

### Patients

This study was approved by the ethical committee of the Affiliated Nanjing Hospital of Nanjing University of Chinese Medicine (Registration No. 2019-LS-ky009). Twelve and four participants were enrolled to explore the distribution of TFV in breast milk after treatment with TAF (25 mg per day) or TDF (300 mg per day), respectively. All participants were between the ages of 20–35 years and had HBeAg-positive chronic HBV infection. Written informed consent was obtained from all participants prior to testing. The inclusion criteria included HBsAg positivity >6 months, HBeAg positivity and HBV DNA concentration >10^6^ IU/ml. The exclusion criteria included combined HCV and HIV infection, severe maternal or fetal illness (malignancy, decompensated liver disease, heart disease, congenital anomaly, etc.), or maternal treatment with drugs or herbal medications with known or uncertain interactions with TAF or TDF. These participants received oral TAF or TDF from 24 to 28 weeks of gestation until the 4th week postpartum. Maternal weight was measured using a digital scale and standing height was measured using a standard stadiometer.

### Sample Collection and Pharmacokinetic Analysis

In the groups of participants administered with 25 mg of TAF daily, the breast milk samples prior to dosing and at 1, 2, 4, 6, 8 h post-dose on the 3rd postpartum day, and prior to dosing and at 1 h post-dose on the 15th and 30th postpartum day were all collected. For participants administered with 300 mg of TDF daily, the breast milk samples prior to dosing and at 1, 2, 4, 6, 8 h post-dose on the 3rd postpartum day, and prior to dosing and at 1 h post-dose on the 7th day were collected. All breast milk samples were obtained by using a breast pump and were stored at −80 °C for further analysis. The concentration of TFV in the breast milk samples were further measured by the UPLC-MS/MS method.

### UPLC-MS/MS Equipment and Conditions

The liquid chromatographic analysis was performed using a Waters UPLC I-Class system comprising of the binary pump, column oven and autosampler (Waters Corp., Milford, MA, United States). The liquid chromatography separation was achieved on a Waters ACQUITY UPLC BEH C18 column (1.7 µm 2.1 × 100 mm) at 40°C. The flow rate was 0.3 ml/min. The mobile phase consisted of solvent A (deionized water containing 2 mM ammonium acetate and 0.1% formic acid) and solvent B (acetonitrile). The elution gradient was as follows: 1% B increased to 50% B from 0.0 to 2.00 min, increased to 98% B from 2.0 to 2.5 min, retuned to initial conditions and then re-equilibrated for 2.5 min. The total run time was 5.0 min.

The UPLC system was interfaced with a Waters Xevo TQ-S triple quadrupole mass spectrometer (Waters Corp., Milford, MA, United States) equipped with an electrospray ionization (ESI) source. MassLynx v4.1 software was used for instrument control, data acquisition and analysis. The mass spectrometer parameters were set as follows: capillary voltage 0.5 kV, source temperature 150°C, desolvation temperature 400°C, desolvation and cone flow rates were set at 800 and 150 L/h, respectively. Mass spectrometry analyses were performed in positive mode and multiple reaction monitoring (MRM) conditions of transitions m/z 288.1→176.2 for TFV(cone voltage at 25 V and collision energy at 24 V) and m/z 294.2→182.1 for TFV-d6 (internal standard, IS, cone voltage at 40 V and collision energy at 24 V).

### Preparation of Stock Solutions, Calibration Standards and Quality Control (QC) Samples

The primary stock solution was prepared by dissolving 10.0 mg of TFV or IS standard in 10 ml of methanol. The stock solutions were diluted with methanol. Blank breast milk mixed standards of TFV were prepared with each set by mixing 5 μL of each standard solution with 45 μL of blank breast milk to obtain final concentrations of 0.5, 1, 5, 10, 50, 100, and 500 ng/ml. QC samples were prepared by employing an independent stock solution to achieve final concentrations of 1,10 and 400 ng/ml in the breast milk. A working solution of IS was daily prepared in acetonitrile at the concentration of 2.5 ng/ml.

### Sample Preparation

An aliquot of 50 μL of breast milk was transferred into a 1.5 ml Eppendorf tube and precipitated with 200 μL of methanol (containing 2.5 ng/ml IS). The mixture was vortexed for 3 min and then centrifuged at 18,800 g for 5 min at 4°C (Thermo, Heraeus Fresco21, United States). The supernatant was injected for analysis.

## Method Validation

The validation of this method was implemented according to the Guidelines for Bio-analytical Method Validation in consideration of linearity, selectivity and sensitivity, precision and accuracy, matrix effect, recovery and other parameters (FDA, 2015).

### Selectivity and Sensitivity

Selectivity was evaluated by comparing the corresponding TFV and IS chromatograms in six independent sources of human breast milk to exclude potential endogenous interference. The lower limit of quantification (LLOQ) was defined as the lowest concentration of analyte. The precision (CV, %) and bias of LLOQ were deemed acceptable if they were within ±20% of each concentration. Each blank breast milk was spiked with TFV at the expected LLOQ.

### Linearity

Linearity was assessed by plotting the peak area ratios of the analytes (Y) to the IS versus the concentrations of TFV (X, 0.5–500 ng/ml), followed by least-squares regression analysis with a 1/X weight.

### Precision and Accuracy

Precision and accuracy were evaluated by analyzing six replicates of QC samples containing known amounts of TFV at three concentration levels (Low, 1 ng/ml; Middle, 10 ng/ml; High, 400 ng/ml). An accompanying standard curve was prepared and determined to obtain the concentration of each sample on the same day. There are six replicates of QC samples at each concentration level in each batch. Intra-day assessment was determined in a single validation batch (n = 6), while inter-day assessment was performed in three batches over three different days. The mean value of bias (%) should be within ±15% of the actual value. The intra- and inter-day precision (CV, %) were required lower than 15%.

### Extraction Recovery and Matrix Effect

The extraction recovery of TFV at the three QC levels was calculated by comparing the peak area of TFV in the extracted breast milk samples with post-extracted breast milk samples spiked with TFV at target concentrations. The matrix effects (ME) of TFV and IS were determined by comparing the peak areas spiked after extraction with those of analytes in reconstitution solution. Corrected matrix factors (MF) by IS were calculated by dividing the ME of TFV by the ME of the IS. The extraction recovery and IS-normalized MF at each QC level were assessed by analyzing six breast milk samples. The corresponding CV (%) were required lower than 15%.

### Stability

The stability of TFV in breast milk was evaluated by analyzing samples at three QC levels under different storage conditions. QC samples were kept at 4°C for 24 h or exposed to room temperature for 6 h to evaluate the pre-preparative stability. QC samples were frozen and thawed for three times or stored at −80°C for 4 weeks to evaluate the storage stability. The extracted QC samples kept in the autosampler at 8°C for 48 h were reinjected to assess the post-preparative stability of the extract. TFV was considered stable when the biases were no more than 15% at the three QC levels.

### Data Analysis

The concentration-time curve of TFV in breast milk samples were plotted and analyzed by GraphPad Prism 5.0 (GraphPad Software Inc., SanDiego, CA, United States). Maximum concentration (C_max_) and the time-to-maximum (T_max_) of TFV in breast milk were determined by direct inspection (Olagunju et al., 2015). The area under the concentration-time curve (AUC_0-t_) was obtained according to the trapezoidal rule. Statistical analysis was performed using SPSS 22.0 (IBM, NY, United States).

## Results

### Method Validation

#### Selectivity and Sensitivity

**FIGURE 1:**
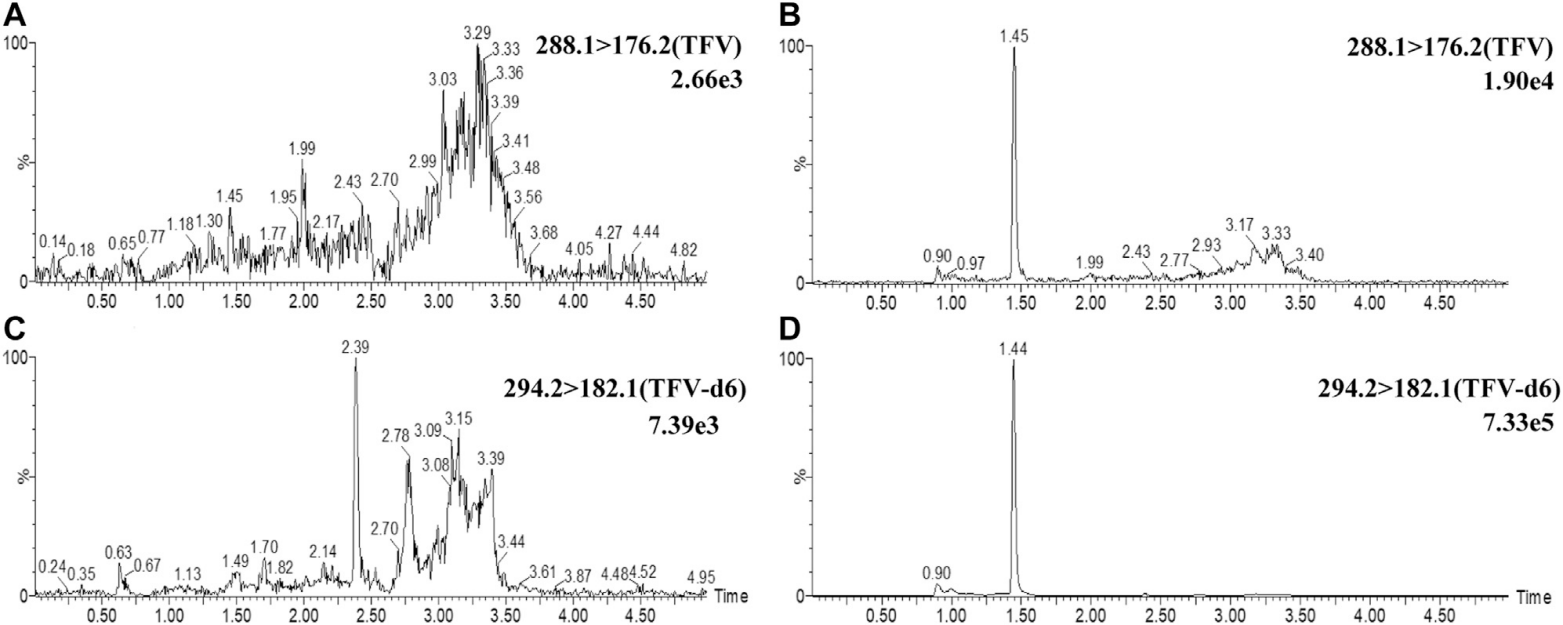
Representative UPLC-MS/MS chromatograms of TFV and IS. Chromatograms of TFV were demonstrated at m/z 288.1→176.2 channel in blank breast milk samples **(A)** and blank breast milk samples spiked with 0.5 ng/ml TFV. **(B)** Chromatograms of IS were demonstrated at m/z 294.2→182.1 channel in breast milk **(C)** and blank breast milk spiked with 2.5 ng/ml IS(D).

Blank breast milk samples from six independent sources were determined, and the typical chromatograms are shown in Figure 1. No endogenous interferences were observed at the peak regions of TFV (1.45 min) and IS (1.44 min). The shapes of the peaks for TFV and IS appeared to be sharp and symmetrical. The signal of noise peaks (S/N) in blank breast milk was less than 20% of that of TFV in spiked samples at 0.5 ng/ml (LLOQ). The S/N ratio at LLOQ was greater than 10. These results demonstrated that the selectivity and sensitivity of this method met the requirements.

#### Linearity

The calibration curves for TFV were exhibited good linearity over the concentration range of 0.5–500 ng/ml, with correlation coefficients (r) higher than 0.99. The LLOQs was 0.5 ng/ml which fulfilled the acceptance criteria of S/N > 10 and bias (%) within ±20%. Meanwhile, bias (%) of other concentrations in calibration curves was all within ±15%. Mean values and standard-deviations of intercepts, determined slopes, and correlation coefficients were respectively as follows (n = 3): 0.00252 ± 0.00185,0.05117 ± 0.00228, 0.99853 ± 0.00107.

#### Precision and Accuracy

The precision and accuracy for TFV at three concentrations were calculated and expressed as a percentage of deviation. The mean values are calculated and expressed in Table 1. The intra- and inter-day precision varied from 1.5 to 3.2% and from 3.4 to 3.8%. The mean values of bias (%) range from 1.7 to 4.2%. At all levels, the results of bias (%) and CV (%) were within the acceptance criteria established by FDA.

**TABLE 1:** Precision and accuracy of TFV in human breast milk.

C_nominal_ (ng/ml)	CV (%)		Bias (%)
	Intra-day	Inter-day	
1	2.4	3.4	2.1
10	1.5	3.8	1.7
400	1.7	3.4	4.2

C_nominal_, theoretical concentration; CV (%), coefficient of variation.

#### Extraction Recovery and Matrix Effect

The extraction recoveries and matrix effects of TFV at the three QC levels were calculated and summarized in Table 2. The mean recoveries of TFV at concentrations of 1, 10, and 400 ng/ml were 110.5, 99.6, and 94.6%, respectively. In addition, the mean corrected MF by IS of six different sources of human breast milk at three concentrations ranged from 98.5 to 105.2%, indicating the matrix effect of human breast milk was negligible for this assay. In summary, the extraction recovery and matrix effect of human breast milk by employing this method were all less than 15% at each level, which is defined to be acceptable.

**TABLE 2:** Recovery rates and matrix effects of the TFV in human breast milk.

C_nominal_ (ng/ml)	Extraction recoveries/%		IS corrected matrix factors/%	
	GM (%)	SD (%)	GM (%)	SD (%)
1	110.5	5.1	105.2	1.5
10	99.6	2.0	99.0	2.5
400	94.6	1.0	98.5	1.0

C_nominal_, theoretical concentration; GM, grand mean; SD, standard deviation.

#### Stability

The stabilities of TFV in human breast milk under different storage conditions were calculated and expressed in Table 3. Bias (%) and CV (%) values were all within 15%, indicating that TFV was satable in human breast milk under typical storage and processing conditions.

**TABLE 3:** Stability evaluation of TFV under different conditions (*n* = 6).

Conditions	Concentrations (ng/ml)		CV (%)	Bias (%)
	C_nominal_	Measurements		
**6 h at room**	1	1.1	2.8	2.6
	10	10.7	1.4	2.4
	400	420.1	1.3	−0.8
**24 h at 4 °C**	1	1.1	2.4	0.9
	10	10.4	3.5	0.1
	400	410.8	0.8	−3.0
**Three freeze-thaw**	1	1.1	3.3	1.7
	10	10.8	1.7	3.2
	400	418.1	0.1	−1.2
**48 h in autosampler at 8 °C (Post-preparation)**	1	1.1	3.6	5.9
	10	10.5	0.2	0.4
	400	419.3	0.9	−0.9
**1 month at -20 °C**	1	1.0	3.9	0.7
	10	9.4	3.1	−6.4
	400	362.2	1.2	−9.4

C_nominal_, theoretical concentration; CV (%), coefficient of variation.

### Distribution of TFV in Breast Milk of Mothers Administered With TAF and TDF

This validated UPLC-MS/MS method was applied to measure TFV concentrations in the breast milk of mothers who were administered with TAF (25 mg per day) or TDF (300 mg per day) from 24 to 28 weeks of gestation until the 4th week postpartum. The distribution of age and BMI values was similar across the mothers administered with TAF and TDF, as shown in Table 4. On the third postpartum day, TFV in the breast milk of twelve mothers administered with TAF reached a median (interquartile range, IQR) Cmax of 101.2 (83.0–123.0) ng/ml at a Tmax of 4.0 (4.0–6.0) h. The AUC0-8 was 755.6 (81.6–894.4) ngh/mL. The median concentrations at 0, 1, 2, 4, 6, 8 h ranged from 83.8 to 101.1 ng/ml. Surprisingly, despite the higher dosage administered in the group of prodrug TDF, the distribution of TFV in the breast milk was relatively lower. The median Cmax of TFV in the breast milk of mothers administered with TDF was only 21.3% of that of mothers treated with TAF, although median Tmax is similar. The AUC0-8 of 150.8 (120.7–180.9) ngh/mL was significantly lower than that in TAF group (*p* < 0.01). The median concentrations at 0, 1, 2, 4, 6, 8 h ranged from 17.3 to 23.2 ng/ml. Overall, the variability of TFV concentrations in breast milk during the dosing interval was relatively small.

**TABLE 4:** Participant characteristics and pharmacokinetic analysis.

	TAF (*n* = 12)	TDF (*n* = 4)
**Age(y)**	28.8 ± 3.0	27.0 ± 2.5
**BMI (kg/m** ^**2**^ **)**	24.3 ± 1.1	23.9 ± 0.8
**Gestational weeks of initial administration**	25.0 (25.0–25.5)	26.5 (25.5–27.0)
**Gestational weeks of delivery**	40.0 (39.0–40.0)	40.0 (39.5–40.5)
**TFV concentration (3**rd **postpartum day)**		
**Prior to dosing**	93.1 (67.7–112.7)	17.3^a^(16.5–18.1)
**1 h (ng/ml)**	90.8 (61.2–108.5)	23.2^b^(20.9–25.4)
**2 h (ng/ml)**	83.8 (69.2–112.7)	18.5^a^(16.8–20.2)
**4 h (ng/ml)**	101.1 (74.9–123.0)	19.0^a^(14.9–23.1)
**6 h (ng/ml)**	96.4 (72.1–109.8)	20.5^a^(14.6–26.5)
**8 h (ng/ml)**	85.9 (73.1–111.7)	16.2^b^(11.6–20.8)
**Cmax (ng/ml)**	101.2 (83.0–123.0)	21.6^a^(16.8–26.5)
**Tmax(h)**	4.0 (4.0–6.0)	4.0 (2.0–0.6.0)
**AUC**_**0-t**_(**ng·h/mL)**	755.6 (581.6–894.4)	150.8^a^(120.7–180.9)

The normality of values was analyzed by Shapiro-Wilk test. Values with normal distribution were expressed as Mean ± SD. Values with non-normality were expressed as median and interquartile range (Q1∼Q3).

a *p* < 0.01.

b *p*< 0.05, compared with TAF group by Mann-Whitney U test.

**FIGURE 2:**
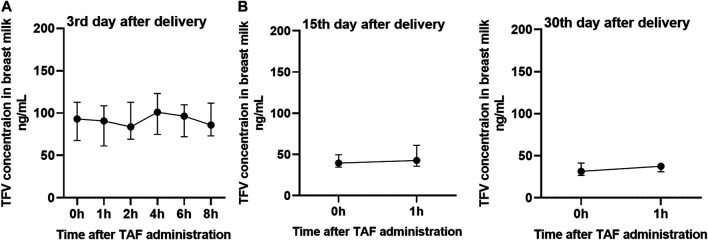
Concentrations of TFV in the breast milk from mothers administered with 25 mg of TAF per day. Concentration-time curve of TFV in breast milk from mothers administered with TAF on the 3rd **(A)**, 15th **(B)** and 30th **(C)** postpartum day. Values are plotted as median (IQR). Details are presented in Table 4.

**FIGURE 3:**
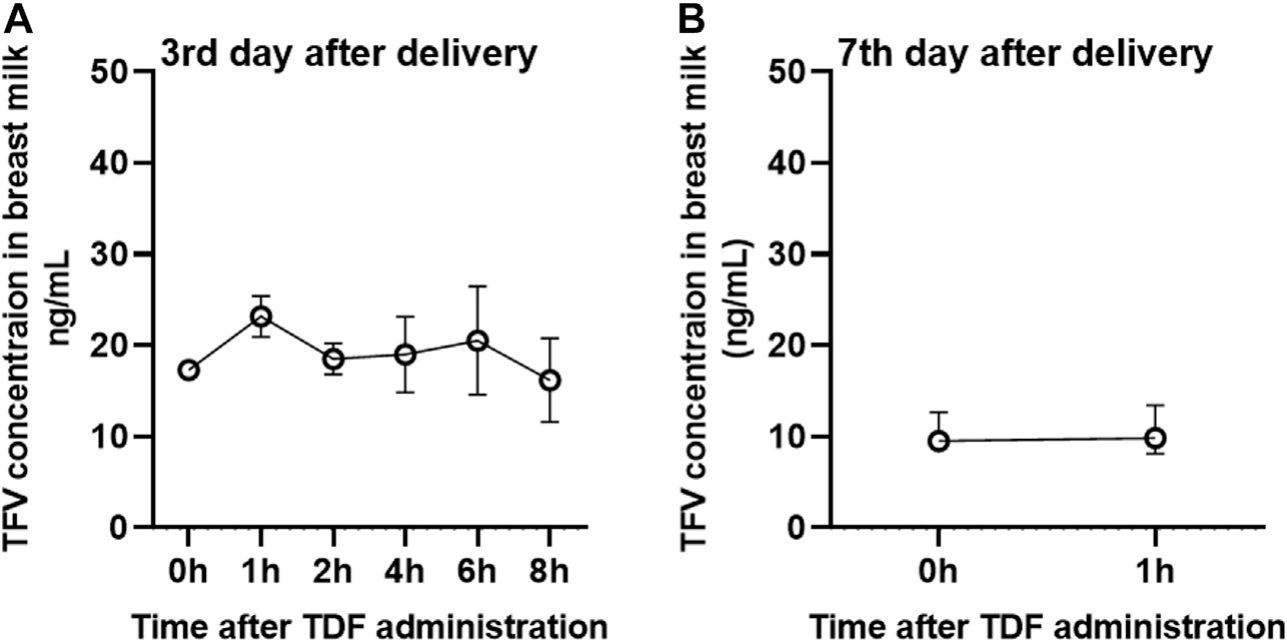
Concentrations of TFV in the breast milk from mothers administered with 300 mg of TDF per day. Concentration-time curve of TFV in breast milk from mothers administered with TDF on the 3rd **(A)** and 7th **(B)** postpartum day. Values are plotted as median (IQR). Details are presented in Table 4.

It is worth noting that the concentration of TFV in breast milk of TAF and TDF groups decreases with the increasing lactation time (Figures 2, 3). Compared to the concentrations of TFV prior to dosing or at 1 h post-dose on the third postpartum day, the median concentrations of TFV in TDF group decreased to less than 10 ng/ml at the same time point on the 7th postpartum day. Besides, the concentrations of TFV in TAF group at the same time points decreased to 42.5 and 47.0% on the 15th postpartum day and was considered to have reached steady state on the 30th postpartum day, as shown in Figure 2.

## Discussion

To the best of our knowledge, this is the first study using the UPLC-MS/MS method to evaluate the distribution of TFV in the breast milk of mothers administered TAF. Previous studies showed that small differences in TFV plasma concentrations occurred when TAF (Markowitz et al., 2014) (25 mg per day) or TDF (Hu et al., 2013) (300 mg per day) was administered. However, the concentrations of TFV in the breast milk of mothers in the TAF administered group were significantly higher than those of the TDF group throughout the entire sampling period, although the oral dose of TAF was lower by more than an order of magnitude compared to TDF (25 vs. 300 mg per day). This inconsistency may be due to the different pharmacokinetic characteristics of TAF and TDF. TDF is almost completely hydrolyzed to TFV in maternal plasma and then transferred into milk by passive or facilitated diffusion down a concentration gradient. TAF is stable in plasma and liposoluble, and compared to TFV, TAF can be distributed in the mammary alveoli much more easily. This means that TAF can be transferred from capillaries to milk-producing lactocytes in the mammary glands, and then be metabolized intracellularly to TFV. Eventually, the metabolized TFV can be easily transferred with maternal proteins, lipids, and immunoglobulins into the milk compartment.

Although the distribution of TFV in the breast milk of mothers taking TAF was unclear before our pilot study, the pharmacokinetics of TFV in the breast milk of mothers treated with TDF has previously been studied. As Benaboud et al. reported, the median maximum and minimal concentrations of TFV in breast milk from five Ivorian mothers treated with a TDF-containing regimen of 300 mg daily since labor until 7 days postpartum were 14.1 and 6.8, respectively (Benaboud et al., 2011). This minimal concentration was similar to the minimal concentration of 9.5 ng/ml on the 7th postpartum day in our study. Palombi et al. observed that the median concentration of TFV in breast milk was 5.0 ng/ml at 1 month after delivery (Palombi et al., 2015). Waitt et al. found that the median maximum concentration of TFV in breast milk from TDF treated mothers after 3 months of delivery was 6.0 ng/ml (Waitt et al., 2018). Overall, there was no obvious difference in the distribution of TFV in the breast milk of mothers receiving TDF treatment in such studies.

In our study, the TFV concentrations decreased significantly for both TAF and TDF. In the first few days after birth, the volumes of colostrum are small, so the alveolar epithelial structure of the breast is porous, which means easy TFV diffusion and high concentrations in breast milk. After several days of breastfeeding, the lactocytes begin to swell and the intercellular junctions narrow, which leads to a gradual decline in the levels of TFV in the milk in the following days postpartum. After birth and breastfeeding begins, accumulated TFV in the mammary glands is gradually excreted in breast milk, which contributes to a time dependent TFV decline in milk, and the concentration of TFV in breast milk ultimately remains stable when milk flow and drug absorption by mammary tissue are stable.

TFV is a dianion at physiological pH and is associated with poor membrane permeability and low oral bioavailability. Although TFV is observed at higher concentrations in the breast milk of mothers orally administered TAF, the TFV absorption of infants through oral breast milk is theoretically not high. Hence, the safety of TAF in the breastfeeding period needs to be further evaluated. Studies on prodrug TAF excretion in breast milk are ongoing to further define the safety and efficacy profile of TAF for lactating women and infants.

## Data Availability

The original contributions presented in the study are included in the article/Supplementary Material, further inquiries can be directed to the corresponding authors.
